# Validity and reliability of the Chinese version of the partners at care transitions measure

**DOI:** 10.1186/s12913-021-07298-z

**Published:** 2021-11-29

**Authors:** La-mei Liu, Ment-ting Liu, Meng-jie Sun, Jia-nan Wang, Bei-lei Lin, Peng Wang, Qiu-fang Li

**Affiliations:** 1 School of Nursing and Health, Zhengzhou University, 100 Science Avenue, High-tech district, Zhengzhou City, 450000 Henan province China; 2 School of Nursing, Philippine Women’s University, Manila, Philippines

**Keywords:** Transition care, Measure, Tool, Validation, Reliability, Patient quality, Safety, Older patients, Chronic diseases

## Abstract

**Background:**

The Partners at Care Transitions Measure (PACT-M) is a measure that assesses the quality and safety of care during the transition from hospital to home from the patient’s perspective. The aim of this study was to examine the psychometric properties of the Chinese version of the PACT-M in Mainland China.

**Methods:**

This was a cross-sectional study. A convenience sample of patients was recruited from three tertiary hospitals affiliated with Zhengzhou University, China. A total of 402 participants were interviewed before discharge, and 306 participants were interviewed one month after discharge from hospital to home using the Chinese version of the PACT-M. The statistical methods used in this study include the critical ratio value, item total correlation, test-retest, Cronbach’s alpha, confirmatory factor analysis and exploratory factor analysis.

**Results:**

The Chinese version of the PACT-M consists of PACT-M_1_ and PACT-M_2_, both of which have two dimensions, the number of items in both parts are consistent with the original English language version. The Cronbach’s alpha values of the PACT-M_1_ and PACT-M_2_ were 0.802 and 0.741, and the test-retest reliability values were 0.885 and 0.837. The item content validity index and scale content validity index values of the PACT-M_1_ and PACT-M_2_ were all 1.0.

**Conclusion:**

The Chinese version of the PACT-M shows acceptable validity and reliability and can be used to assess the quality and safety of transitional care from hospital to home from the patient’s perspective in mainland China.

## Background

Care transition is defined as a series of activities to ensure the coordination and continuity of healthcare for patients who move between different locations or levels of care within the same location [1, 2]. This is most typical among elderly patients with chronic diseases, who have more complex conditions and varying needs for health care during transitions [3]. Transition locations often include (but are not limited to) hospitals, subacute and postacute nursing facilities, the patient’s home, primary and specialty care offices, and long-term care facilities [4]. At present, China’s basic medical and health service network is essentially complete, but in the actual operation of the basic medical service network, because of a lack of awareness, the full capacity of medical services is not utilized; thus, most transitions are from hospital to home [5]. Ideal transitional care should be patient-oriented and based on a comprehensive care plan, which requires considerable and accurate cooperation between various health care providers and clinical teams, including the hospital, primary care team, social services, home care clinicians, and community health professionals [6–8]. In addition, family caregivers, including spouses, children, or other family members, are the primary providers of services to elderly patients with chronic diseases, and their active participation in transitions is essential [9, 10].

The rapid aging of China’s population and the high prevalence of chronic diseases among the elderly are creating serious challenges for China’s economy, social development and health care. The seventh national census shows that the current population of people aged 60 and above in China is 264 million, accounting for 18.7% of the total population, with 190.6 million people aged 65 and above, accounting for 13.5% [11]. There is a high prevalence of chronic diseases in the elderly, with 3/4 of elderly persons suffering from one or more chronic diseases [12]. Chronic diseases are often long-lasting, recurring, and persistent; therefore, patients frequently need to move between the hospital, community, and home to receive different levels of health care. The transition of patients from hospital to home is particularly critical for older patients, especially those with complex care needs [7, 13, 14]. Several studies have also shown that patients with chronic conditions are especially vulnerable and have a greater need for health care during transitions, but they are poorly managed during this time [6, 15, 16], which increases the incidence of adverse outcomes during transfers between settings or providers [6, 16, 17]. Common adverse events during transitions include unplanned readmission to the hospital within the first month after discharge [18–20], medication errors [21, 22], and even mortality [23]. These adverse events are often related to insufficient clinical information and poor communication between the hospital and primary care services [6, 24, 25].

High-quality transition care is an important way to improve patient health outcomes. The 2006 Institute of Medicine report “Performance measurement: accelerating improvement” identified patient-centered transitional care from hospital to home as 1 of 3 priority areas for performance measurement [26]. The Joint Commission and The National Quality Forum (USA) [10, 27, 28] also place great emphasis on high-quality care transition. Patients’ experiences are an important element in the measurement of care transition quality [29, 30]. Several studies have shown that patient experience is a reliable indicator of health care quality and positively correlates with patient safety and clinical efficiency [31, 32]. Current tools for assessing the quality of care transition from the patient’s perspective include the Care Transitions Measure (CTM) [33], the Questionnaire to Measure Older People’s Experience of the Transition Care Program [34], the Partners at Care Transitions Measure (PACT-M) [7, 16], the Discharge Care Experiences Survey (DICARES) [35], the Patient Continuity of Care Questionnaire (PCCQ) [36] and the Nijmegen Continuity Questionnaire (NCQ) [37], of which the CTM is the most common tool used to evaluate the quality of care transitions from hospital to home. It has been translated into Chinese by Bakshi, A. B. et al and Cao, X. et al for use in Singapore and mainland China, and its reliability and validity have been widely validated [24, 29, 38, 39]. Although the CTM is a validated instrument for assessing transition quality relative to information transfer, patient empowerment, patient and caregiver preparation, it focuses only on the immediate postdischarge period and does not include safety-related components [16]. The Questionnaire to Measure Older People’s Experience of the Transition Care Program is part of the National Evaluation of the Transition Care Program (NETCP) commissioned by the Australian Government Department of Health and Aging and evaluates patients’ experiences of care transition from the hospital to nonacute care settings (such as a nursing home) in three dimensions: rehabilitation, continuity and participation. The questionnaire is useful for understanding patients’ experiences in transitional care and for continuous improvement of transitional care [34]. However, it focuses only on the transition of patients from hospitals to nonacute facilities, and its internal consistency is relatively low (Cronbach’s alpha 0.65). There are few studies on this questionnaire and a lack of extensive validation of its reliability. The DICARES was developed by Boge RM et al. based on the literature and is a 10-item questionnaire to assess elderly patients’ experiences with hospital discharge processes and the period after hospitalization. The reliability and validity of the DICARES were also verified, but some of the items in the questionnaire were derived from the CTM, and the authors reported a low response rate (64.4%). There are no studies on this questionnaire and a lack of extensive validation of its reliability [35]. The PCCQ was adapted by Heather's team in Canada based on the Heart Continuity of Care Questionnaire (HCCQ), which is a tool specifically designed to assess transitional care in cardiac specialties, and the PCCQ retains its measurement strengths to some extent and is relevant to the assessment of transitional care for patients with cardiac disease. However, the applicability of this questionnaire to study subjects other than those with cardiac disease is not fully understood, and its widespread use needs further validation [36]. The NCQ was developed by Uijen et al. to assess continuity of care in primary and secondary care from the patient's perspective. It also has good reliability and validity. However, it focuses mainly on continuity of care for patients during the transition between primary and secondary health care [37]. The PACT-M was developed based on the UK health system to assess the quality and safety of care transitions from the patient’s perspective. It consists of 17 items in two parts, the PACT-M_1_ (9 items) and PACT-M_2_ (8 items). The PACT-M_1_ captures the immediate postdischarge period, and the PACT-M_2_ is used to assess the longer-term experience of managing health and care at home. Its scope and content are more comprehensive than those of other scales. Because the PACT-M includes patients’ experiences of care transition in the immediate postdischarge period and their longer-term experiences of managing health and care at home, it can elicit more comprehensive and integrated responses regarding the quality of care transitions.

The PACT-M has been demonstrated to be a valid tool in the UK. However, whether this measure can assess the quality and safety of transitional care for elderly patients with chronic diseases in China needs to be further tested. Therefore, the purpose of our study was to examine the psychometric properties and test the reliability and validity of the Chinese version of the PACT-M.

## Method

### Samples

This was a cross-sectional study using a convenience sampling method. For the purposes of this analysis, we estimated our desired sample size following the sample size strategy outlined in Devellis [40], which states that the ideal sample size for factor analysis should be 15:1 or 20:1 [41]. The PACT-M includes 17 entries, and we calculated a sample size of 408 based on a 20:1 ratio sample size to entries, taking into account a 20% missing visit rate. Participants were recruited while they were in the hospital over a period of six months. To facilitate patient recruitment, we based the study on 3 university-affiliated tertiary-level hospital wards caring for elderly patients with chronic diseases, including cardiovascular, respiratory, endocrine, neurology, oncology, urology and other wards. The survey was conducted in two stages: the first stage used PACT-M_1_ to conduct a face-to-face survey when patients were about to be discharged from the hospital, and the second stage used PACT-M_2_ to conduct a telephone interview with patients who had participated in the first stage of the survey 1 month after discharge. A total of 420 patients with chronic disease participated in this study. The inclusion criteria for patients were as follows: (1) the patients were chronic patients aged 65 years or older; (2) the patients could be contacted by telephone after discharge; (3) the patients received hospital care in the areas of general medicine, geriatric medicine or oncological medicine and were discharged to home rather than long-term care facilities; (4) the patients had no obvious cognitive or language disabilities; and (5) the patients were willing to participate in the survey. To avoid bias from different interviewers during the survey, all interviewers were trained to discuss any questions or concerns before administering the PACT-M. In the first stage, we used the PACT-M_1_ to conduct a face-to-face survey, providing prompt answers to respondent questions. When a patient could not fill out the questionnaire by themselves, the patient dictated the answers to the investigator, who filled it out for them. Among the 420 participants, there were 12 patients who dropped out midway and 6 patients who did not want to continue filling out the questionnaire (95.71% recovery rate). In the second stage, we used the PACT-M_2_ to follow up with the respondents by telephone. Sixty participants withdrew from the study, including 45 who did not respond to multiple phone calls and 15 who did not want to continue to participate because their condition had worsened, and 306 people completed the PACT-M_2_ (72.86% recovery rate).

### The measure

#### The Partners at Care Transitions Measure

We used the original version of the PACT-M to assess patient experience, quality and safety of care transition. The PACT-M includes 17 items, and each item was rated on a 5-point Likert scale from 1 to 5 (“strongly disagree” to “strongly agree”), with higher scores indicating better quality of care during transition. There is also a “not applicable” option, but it was not included in the total score. The authors of the original scale measured that its Cronbach’s alpha coefficients for the two parts of the scale were 0.84 and 0.92, respectively. In addition, demographic characteristics such as sex, age, marital status, illness, medical insurance, and other index conditions were also collected.

### Translation procedure

Authorization was obtained from the authors of the original scale, and the Brislin translation model was used to translate the PACT-M [42]. First, two translators with bilingual proficiency (a nursing graduate student and a nursing doctoral student) independently translated the scale. Our research team and two translators compared and discussed the differences between the two translated versions and then created the Chinese version of the PACT-M. Second, two other translators (a Chinese professor of English linguistics with more than 3 years of experience in English-speaking countries and a second graduate student in nursing science in the USA) translated the Chinese version back into English. Finally, our research team compared the backwards translation with the original English version, provided feedback and confirmed the conceptual and literal equivalence of the Chinese version.

Moreover, we recruited 30 elderly patients with chronic diseases who met the inclusion criteria in the geriatric ward of university-affiliated hospitals and conducted a presurvey at the time of imminent discharge and 1 month after discharge. The results supported the readability, comprehensibility and cultural adaptation of the Chinese version. All participants understood the items easily and took 15~28 minutes to complete the questionnaire.

### Data collection

Seven experts were invited to evaluate the content validity of the Chinese PACT-M. These experts included two professors specializing in chronic disease management, one professor specializing in mental health, two associate professors in geriatric care, and a professor and associate professor with experience in questionnaire development. Based on the experts’ professional theoretical knowledge and clinical work experience, they were asked to comment on the clarity of expression, language conventions, cultural background and content relevance of each item of the scale, and the item content validity index (I-CVI) and scale content validity index (S-CVI) were then calculated to evaluate the content validity using a four-point scale ranging from 1 (not relevant) to 4 (highly relevant) [43].

A survey team was formed by three postgraduate students who had undergone uniform training. After obtaining consent from the relevant hospital departments, department heads and patients, the patients were first informed of the purpose and importance of this study, and each patient was asked to sign an informed consent form. The PACT-M_1_ was then used to survey patients who were about to be discharged from the hospital and met the inclusion criteria. To protect patient privacy, patients completed the questionnaire anonymously but were required to provide their contact information for follow-up. The investigator used uniform instructional language to explain the purpose of the survey, the method of completing the questionnaire and precautions to the respondents. The questionnaires were distributed and collected within the same visit. After each patient completed the questionnaire, the investigator performed a preliminary check to ensure that the questionnaire was complete. If there were any missing answers, the investigator communicated with the patient to determine the reason that they had not answered the questions. If the missing answers were because the patient had missed the questions, the investigator asked the patient to complete the information. If the patient did not know how to answer, the investigator explained the meaning of the question to the patient and then allowed the patient to complete the questionnaire.

One month after surveying with the PACT-M_1_, patients who participated in the first survey were followed up by telephone using the PACT-M_2_, and if they did not answer, they were considered to have dropped out halfway. In addition, we performed test-retest reliability testing of both the PACT-M_1_ and PACT-M_2._ Polit, D. F.’s [44] study showed that the time interval for retesting is usually 1 or 2 weeks; thus, we selected 30 patients from 402 and 306 patients for a second telephone survey two weeks after the first round to check the retest reliability of the questionnaire. Patient selection began with questionnaire number 1 and continued until 30 patients were recruited. Excluding patients who consistently did not answer the phone during the survey, all surveys were conducted between August 1, 2020, and December 30, 2020.

### Data analyses

The returned questionnaires were numbered uniformly, the data were entered by two people, and EpiData 3.1 software was used for systematic logical error checking. The data were statistically analyzed using SPSS 21.0 and AMOS 25.0. Categorical variables were statistically described as frequencies and percentages; continuous variables that conformed to a normal distribution were statistically described as the means and standard deviations; and the scale response rate was used to test patients’ acceptance of the Chinese version of the PACT-M.

We used the critical ratio value (CR) and item total correlation (ITC) to calculate the item discriminatory power. We set the respondents with total scores in the top 27% as the high group and the respondents with total scores in the bottom 27% as the low group and then conducted an extreme group comparison using a t-test. The ITC was calculated using Spearman correlation analysis. The ITC values were higher than 0.30, and statistical significance (*P*<0.001) was considered to indicate desirable discriminating power [45].

We estimated the test-retest reliability using Pearson correlation analysis with a two-week interval between evaluations. We used Cronbach’s alpha value to estimate the internal consistency of the Chinese version of the PACT-M.

We calculated the I-CVI as the ratio of the number of “highly relevant” and “quite relevant” expert-opinion responses to the number of experts. We calculated the S-CVI as the average of the I-CVI values for all items rated as either “highly relevant” or “quite relevant” [46].

We used exploratory factor analysis (EFA) with principal component extraction and varimax rotation to identify the factor structure of the Chinese version of the PACT-M. The Kaiser-Meyer-Olkin (KMO) and Bartlett's test were used to test whether the data were suitable for EFA. Based on the results of EFA, confirmatory factor analysis (CFA) was performed using the maximum-likelihood method to obtain the structural equation model.

### Ethical statement

First, we received permission from the original authors to translate the PACT-M into Chinese and use it. Second, the study was approved by the Zhengzhou University ethics committee in China (approval number: ZZUIRB2021-78) and followed the Declaration of Helsinki Ethical Principles for Medical Research Involving Human Subjects. Finally, consent was obtained from all participants, including the experts, translators, and patients, and the patients were guaranteed confidentiality, anonymity and the right to withdraw from the study at any time.

## Results

### Participant characteristics

In total, 402 people completed the PACT-M_1_ (response rate 95.71%), and 306 people completed the PACT-M_2_ (response rate 72.86%). The mean age of the sample was 74.4 years (range: 65–98). A total of 46.8% of the participants were male, and most of the respondents were married (96.7%). A total of 69.4% of the respondents were educated at the junior high school level and below. Almost all respondents had health insurance (94.8%). Regarding the types of diseases, 29.1% of respondents suffered from hypertension, 16.9% from heart disease, 10.4% from diabetes mellitus, 9.5% from chronic lung disease, 8.2% from stroke, 4.0% from cancer, 1.2% from kidney disease, and 20.6% from other diseases. Further demographic characteristics are presented in Table 1.

**Table 1:** Participant characteristics (*n* = 402)

Variable		*N*=402	Percent (%)
**Sex**	Female	214	53.23
	Male	188	46.77
**Age**	65~	225	55.97
	75~	141	35.07
	85~	32	7.96
	95~	4	0.10
**Marital status**	Married	385	95.77
	Single/divorced/widowed	17	4.23
**Education level**	Junior high school and below	279	69.40
	High school and college	98	24.38
	Undergraduate	18	4.48
	Graduate student and above	7	1.74
**Household income per month (Yuan)**	<1000	84	20.90
	1000~	125	31.09
	3000~	102	25.37
	5000~	91	22.64
**Residence**	City	168	41.79
	Town	73	18.16
	Rural	161	40.05
**Preretirement occupation**	Worker	112	27.86
	Farmer	181	45.02
	Enterprise (business) unit	22	5.47
	Individual household	58	14.43
	Military	14	3.48
	Medical and nursing personnel	11	2.74
	No fixed work	4	1.00
**With or without a caregiver**	With	352	87.56
	Without	50	12.44
**If there is a caregiver, the caregiver is**	Spouse	136	33.83
	Children	191	47.51
	Other	75	18.66
**Religion**	With	59	14.68
	Without	343	85.32
**Ethnic group**	Han Chinese	374	93.03
	Other	28	6.97
**Medical insurance**	Yes	374	93.03
	No	28	6.97
**Living situation**	Living alone	39	9.70
	Living with spouse only	135	33.58
	Living with more than one person	228	56.72
**Disease type**	Chronic lung disease	38	9.45
	Cancer	16	3.98
	Diabetes mellitus	42	10.45
	Heart disease	68	16.92
	Kidney disease	5	1.24
	Stroke	33	8.21
	Hypertension	117	29.10
	Other disease	83	20.65

### Results of the item analysis

The results of the CR test showed that the differences between items in both parts were statistically significant (*P*<0.001). The correlation coefficients between items in both parts and the total score of the scale were 0.493~0.622 and 0.450~0.669 (*P*<0.001), respectively, which were >0.40; thus, no items were deleted.

### Results of EFA

The KMO values of the Chinese PACT-M_1_ and PACT-M_2_ were 0.846 and 0.791, respectively, and Bartlett’s test of sphericity λ^2^ values were 792.038 and 404.250, respectively (*p*<0.001), according to N Shrestha's [47] study, KMO values between 0.6 and 1.0 indicate that the sampling is adequate, and the significance value of Bartlett’s test was <0.001, which indicated that factor analysis may be worthwhile for the dataset. In this study, the data were orthogonally rotated by principal component analysis and the maximum variance method, and 2 common factors were extracted for the Chinese PACT-M_1_ and PACT-M_2_. The amounts of variance explained by the 2 common factors of PACT-M_1_ were 39.036% and 13.048%, with a cumulative variance contribution of 52.516%. The amounts of variance explained by the 2 common factors of PACT-M_2_ were 36.397% and 14.261%, with a cumulative variance contribution of 50.658%.

Based on our EFA results, the Chinese version of the PACT-M contains two parts, similar to the original version, but we extracted two factors from each part, unlike in the original version. We named factor 1 of the PACT-M_1_ “Perceived health management support at the hospital” and factor 2 “Received information and support at the hospital”, and we named factor 1 of the PACT-M_2_ “Perceived health management support at home” and factor 2 “Home health management”. Factor loading analysis was further applied to the variance maximum orthogonal rotation method, and the results are shown in Table 2.

**Table 2:** Factor loading of the Chinese version of the PACT-M

Factor loadings for each PACT-M1 item	Factor 1	Factor 2
**Factor 1: Perceived health management support at the hospital**		
1. I felt I could ask staff questions about what will happen after going home, A1.	**0.597**	0.129
2. Before leaving the hospital, I was confident I understood how to manage my medication, A2.	**0.687**	0.089
6. While I was in hospital, there was someone who I could talk to if I was worried, A6.	**0.467**	0.439
7. Before leaving the hospital, I felt confident about what to do if my health became worse at home, A7.	**0.736**	0.246
8. I feel that my concerns regarding my health were addressed before I went home, A8.	**0.736**	0.217
9. I felt prepared to be at home, A9.	**0.630**	0.102
**Factor 2: Received information and support at the hospital**		
3. While I was in the hospital, staff helped me to prepare for things that I might find difficult when I go back home (such as walking, cooking, showering, shopping, or being in pain), A3.	0.265	**0.736**
4. Before leaving the hospital, I understood how to get help (or support) from my community services (e.g., doctors, nurses, and home care staff), A4.	0.060	**0.81**
5. Before leaving the hospital, I knew what arrangements had been made to support me at home (for example, home care or community care visits), A5.	0.169	**0.763**
**Factor loadings for each PACT-M2 item**		
**Factor 1: Perceived health management support at home**		
4. I feel I have the support I need from community health services (e.g., doctors, nurses, and home care staff), B4.	**0.567**	0.222
5. I feel confident about managing my health at home, B5.	**0.764**	0.061
6. I feel that there is someone I can talk to about my worries (for example, health care staff or my family), B6.	**0.799**	-0.001
7. I know what to do and who to contact if my health gets worse, B7.	**0.649**	0.281
8. I feel I can now manage my care safely at home, B8.	**0.608**	0.157
**Factor 2: Home health management**		
1. I know who to contact if I have any questions about my health and healthcare, B1.	0.108	**0.649**
2. I know how to manage my medications, B2.	0.030	**0.785**
3. I have the necessary support to manage everyday activities (e.g., cooking, cleaning, buying food, showering, walking, and dressing), B3.	0.357	**0.621**

### Results of CFA

**Fig. 1:**
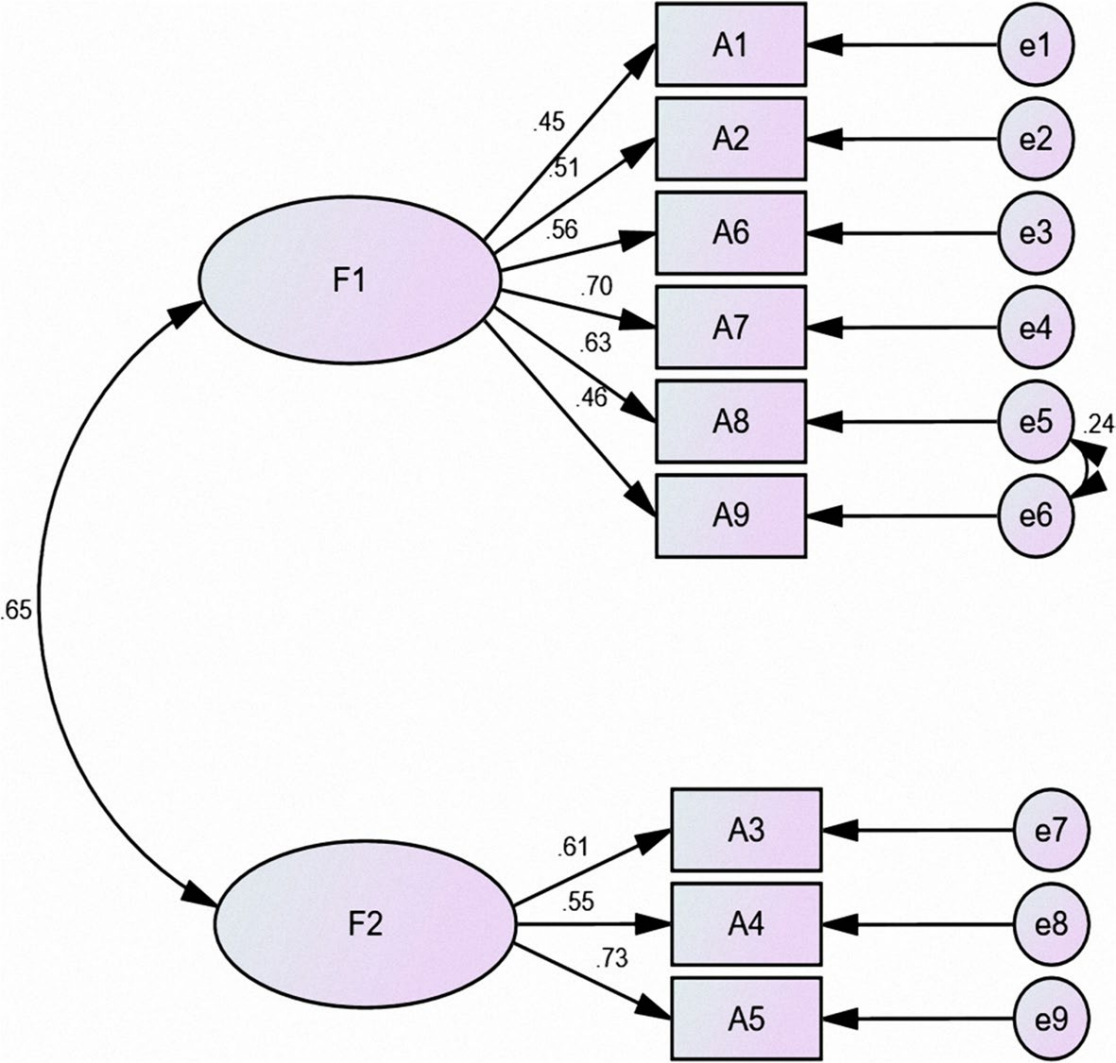
PACT-M1 structural equation model

**Fig. 2:**
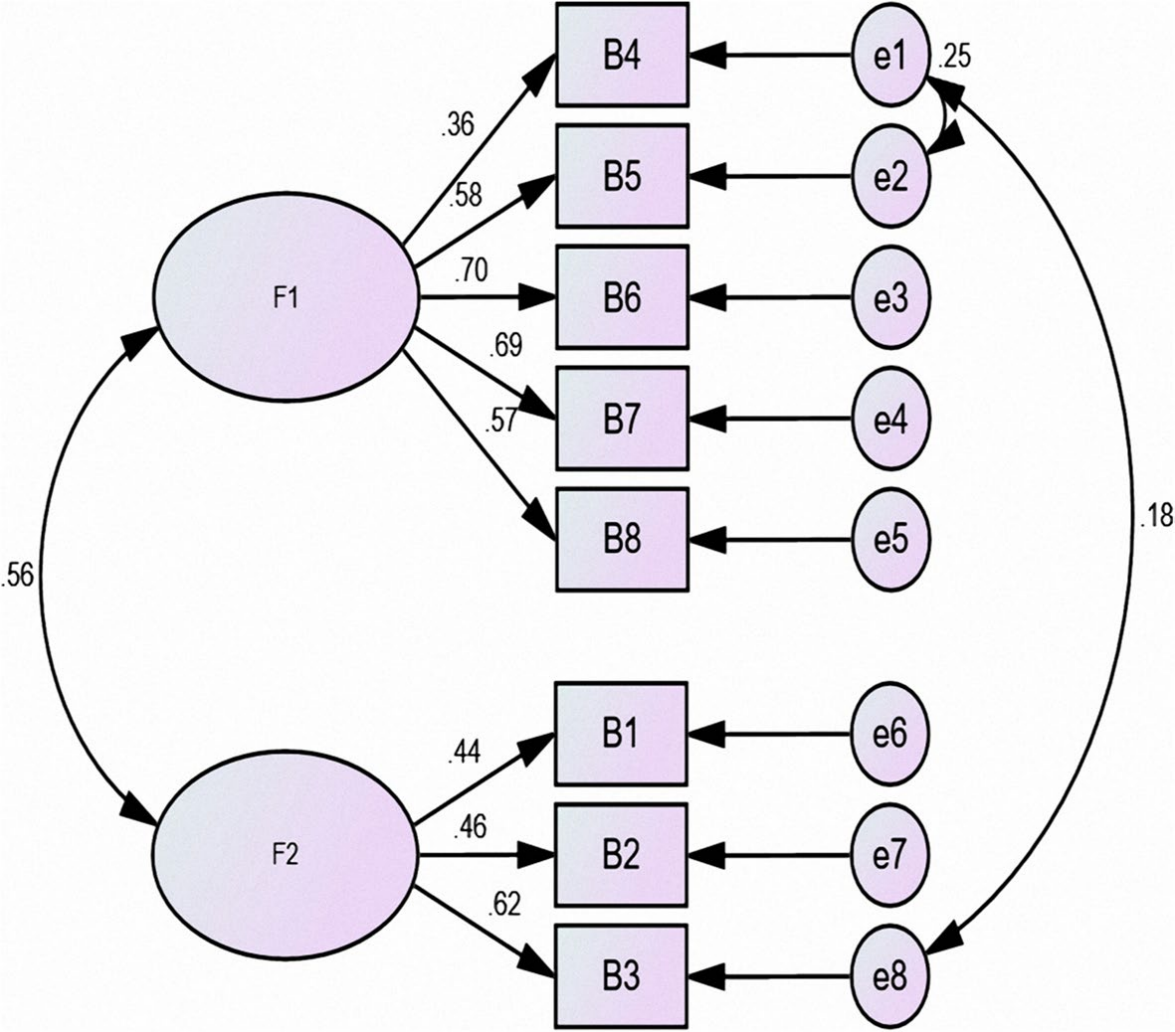
PACT-M2 structural equation model

CFA was performed using the maximum-likelihood method to obtain the structural equation model. The results are shown in Figure 1 (PACT-M_1_ structural equation model) and Figure 2 (PACT-M_2_ structural equation model), and the fit indices are shown in Table 3.

**Table 3:** Chinese version of the PACT-M validated factor analysis fit indices

Indicator	Fit criteria	PACT-M_1_ score	PACT-M_2_ score
Cardinality/degree-of-freedom ratio(χ2/df)	<3.0	1.679	1.483
Root mean square error of approximation (RMSEA)	<0.05	0.041	0.040
Goodness-of-fit index (GFI)	>0.9	0.977	0.980
Comparative fit index (CFI)	>0.9	0.975	0.980
Normative fit index (NFI)	>0.9	0.942	0.942
Relative fit index (RFI)	>0.9	0.917	0.904
Nonnormalized fit index (TLI)	>0.9	0.965	0.967
Adjusted goodness-of-fit index (AGFI)	>0.9	0.959	0.958
Value-added fit index (IFI)	>0.9	0.976	0.980

### Reliability of the Chinese version of the PACT-M

The Cronbach’s α of the Chinese versions of the PACT-M_1_ and PACT-M_2_ were 0.802 and 0.741, respectively, the Cronbach’s α values for the two dimensions of PACT-M_1_ were 0.757 and 0.702, and the Cronbach’s α values for the two dimensions of PACT-M_2_ were 0.739 and 0.572. The Spearman-Brown split-half coefficients were 0.748 and 0.626 for PACT-M_1_ and PACT-M_2_, respectively. The Spearman-Brown split-half coefficients for the two PACT-M_1_ dimensions were 0.735 and 0.712, and the Spearman-Brown split-half coefficients for the two PACT-M_2_ dimensions were 0.705 and 0.521. The test-retest reliability values were 0.885 and 0.837. The retest reliability values of the two PACT-M_1_ dimensions were 0.826 and 0.913, and the retest reliability values of the two PACT-M_2_ dimensions were 0.780 and 0.654. Both the I-CVI and the S-CVI of PACT-M_1_ and PACT-M_2_ were 1.00.

## Discussion

This study explored the reliability and validity of the Chinese version of the PACT-M. This was the first cultural commissioning of the PACT-M in China. Although the PACT-M was developed focusing on the health care system in the UK, after our translation and cultural adaptation, we found that the PACT-M had good reliability and validity and that patients could understand the content of the questionnaire and complete it within 15-28 minutes. It can be used as a metric for discharge and continuity of care quality and to identify areas for improvement of services targeted to care transitions.

In the item analysis, we found that the statistical values of each item in both parts reached significant levels, and the difference was statistically significant (*P*<0.001), indicating that the Chinese version of the PACT-M had good discrimination. The correlation coefficients between each item and the total score were 0.493~0.622 and 0.450~0.669, which were both >0.4, indicating that each item could adequately measure the quality and safety of care during transitions [45].

Regarding the validity analysis, Polit et al [48] suggested that the content validity index at the item level should be 0.9 or higher to be considered good. In our study, both the I-CVI and the S-CVI were 1.0, indicating that the Chinese version of the PACT-M had good content validity.

In EFA, the cumulative variance contributions of the common factors were at least 40%. Each item must have a high absolute loading value on one of the common factors for the measure to be considered to have good structural validity [49]. In this study, the PACT-M_1_ and PACT-M_2_ items were the same as in the original version, and two common factors were extracted from the two parts of the Chinese version of the PACT-M using EFA. These factors had cumulative variance contribution rates of 52.516% and 50.658%, which were not consistent with the original single-dimension version [16]. The difference may be attributable to the different healthcare systems in mainland China and the UK. In the UK, family physicians provide health management for patients after they return home from the hospital. However, in mainland China, patients’ health is usually managed by themselves or their caregivers after discharge. Patients need to visit community health centers if they want to receive relevant health services. Therefore, there are differences in patient perceptions and the health services that they have received. In the PACT-M_1_, factor 1 included items 1 (patient involvement), 2 (medication management), 6 (psychological and social support), 7 (prediction and preparation for emergencies or deterioration), 8 (ready for discharge), and 9 (ready for home). We named factor 1 “Perceived health management support at the hospital”. Item 6 loaded similarly on factors 1 and 2, but “While I was in the hospital, there was someone who I could talk to if I was worried” and “Perceived health management support at the hospital” were more professionally relevant and were ultimately assigned to factor 1. Factor 2 included items 3 (discharge arrangements), 4 (coordination with other health care professionals), and 5 (information and guidance to the patient or family). We named factor 2 “Received information and support at the hospital”. In the PACT-M_2_, factor 1 included items 4, 5, 6, 7, and 8, and we named factor 1 “Perceived health management support at home”. Factor 2 included items 1, 2, and 3, and we named factor 2 “Home health management”. Overall, EFA showed that the factor loadings of each item were greater than 0.4, and CFA also showed that the fit indices were good, indicating that the actual survey data of the Chinese version of the PACT-M fit the structural equation model well. These results suggest that the Chinese version of the PACT-M has good structural validity.

In reliability analysis, we found that the Cronbach’s α values of the C-PACT-M_1_ and C-PACT-M_2_ were 0.802 and 0.741; the Spearman–rown split-half coefficients were 0.748 and 0.626 for the PACT-M_1_ and PACT-M_2_, respectively; and the Spearman-Brown split-half coefficients between the dimensions were 0.735 and 0.712 and 0.705 and 0.521, respectively. These findings are consistent with the findings of the original authors [16]. A measure is reliable if its Cronbach’s α value exceeds 0.70 [50]. The reliability of the Chinese version of the PACT-M was generally satisfactory, although the second dimension (self-care experience) of the PACT-M_2_ had a low internal consistency of 0.572 and a Spearman-Brown split-half coefficient of 0.521. The low reliability may be related to the small number of entries [24]. The test-retest reliability of the two parts of the Chinese version of the PACT-M were 0.885 and 0.837, respectively, which demonstrated good stability of these two measures over time. A test-retest reliability value over 0.80 indicates good reliability [51]. However, the possibility that some respondents’ memory of their first response led to higher retest reliability should also be considered, and longer time intervals and larger sample sizes should be included in subsequent studies.

There are several limitations in our study. First, we used a convenience sampling method, and the participants were recruited from three general tertiary-level hospitals in Zhengzhou, China, which might weaken the representativeness of the sample and limit the generalizability of our findings. Further study is needed to ascertain the applicability of the scale to elderly patients with chronic diseases in other cities. Second, we excluded patients with psychiatric disorders, cognitive impairment, or inability to communicate because of vision or hearing impairment. Thus, the applicability of the scale to this group of patients remains unclear, and future studies may be conducted specifically to analyze the applicability of the scale to patients with difficulties communicating. Third, we did not perform criterion-related validation of the constructs in our Chinese version of the PACT-M, which is needed in future research.

Despite these limitations, our findings indicate that the Chinese version of the PACT-M is a reliable and valid instrument for assessing the quality and safety of care during the transition from hospital to home in mainland China. Although the Cronbach’s α value of factor 2 of the PACT-M_2_ was suboptimal, it exhibited favorable test-retest reliability and construct validity. Thus, the Chinese version of the PACT-M is an effective performance measurement tool when the sample size is large enough to compensate for its suboptimal reliability or the reduced response burden is a concern. Moreover, the development of the Chinese version of the PACT-M has several important implications. First, health care providers should consider the quality and safety of care during transitions for elderly patients with chronic diseases, which they can evaluate using the PACT-M. Second, the PACT-M may be useful as a potential screening instrument to detect patients who need help, and it can be used by health care providers to provide targeted care to improve patient health. Third, the PACT-M not only assesses the quality and safety of care upon preparation for discharge but can also assess the experience of managing care at home, facilitating assessment of patient experiences across a broader transition time span. Finally, the PACT-M can be used to assess the effectiveness of interventions, which may help improve the quality and safety of care for patients with chronic disease.

## Conclusion

The results of our study suggest that the Chinese version of the PACT-M shows acceptable validity and reliability and can be used to assess the quality and safety of care during the transition from hospital to home from the patient’s perspective in mainland China. Our version of the PACT-M has slightly different dimensions than the English version, which suggests that tools may need to be modified when used in different countries or in different cultures. Given the limitations of this study, future research should focus on expanding the sample size and further validating the applicability of the scale in elderly patients with chronic diseases. In addition, future research should explore the validity of the PACT-M by comparing the PACT-M with other similar scoring systems.

## Data Availability

The datasets used and analyzed during the current study are available from the corresponding author upon reasonable request.

## Abbreviations

PACT-M: Partners at Care Transitions Measure
CTM: Care Transitions Measure
DICARES: The Discharge Care Experiences Survey
PCCQ: The Patient Continuity of Care Questionnaire
NCQ: The Nijmegen Continuity Questionnaire
NETCP: The National Evaluation of the Transition Care Program
I-CVI: Item content validity index
S-CVI: Scale content validity index
CR: Critical ratio value
ITC: Item total correlation
EFA: Exploratory factor analysis
KMO: The Kaiser-Meyer-Olkin
CFA: Confirmatory factor analysis

## References

[1] Eric A, Coleman C, Boult. (2003). Improving the Quality of Transitional Care for Persons with Complex Care Needs : Position Statement of The American Geriatrics Society Health Care Systems Committee. J Am Geriatr Soc.

[2] Naylor MD (2000). A decade of transitional care research with vulnerable elders. J Cardiovasc Nurs.

[3] Baxter R, O'Hara DJ, Murray DJ, Sheard DL, Cracknell DA (2018). Partners at Care Transitions - exploring healthcare professionals' perspectives of excellence at care transitions for older people: a study protocol. BMJ Open.

[4] Coleman EA (2010). Falling through the cracks: challenges and opportunities for improving transitional care for persons with continuous complex care needs. J Am Geriatr Soc.

[5] Xiliang LI, Zuo Q, Liu S (2013). Study on the path how to establish the brand of primary medical care services in the elderly population of the community. Med Res Educ.

[6] Naylor MD (2012). Advancing High Value Transitional Care: The Central Role of Nursing and Its Leadership. Nurs Adm Q.

[7] Oikonomou E, Chatburn E, Higham H, Murray J, Vincent C (2019). Developing a measure to assess the quality of care transitions for older people. BMC Health Serv Res.

[8] Burke RE, Kripalani S, Vasilevskis EE, Schnipper JL (2013). Moving beyond readmission penalties: Creating an ideal process to improve transitional care. J Hosp Med.

[9] Coleman E, Parry MC, Chalmers S, Min SJJAoIM. (2006). The care transitions intervention: results of a randomized controlled trial. Arch Intern Med.

[10] Coleman EA, Berenson RA (2004). Lost in transition: challenges and opportunities for improving the quality of transitional care. Ann Intern Med.

[11] Bureau SS (2021). The Seventh National Population Census Bulletin (No. 5).

[12] Limin W, Zhihua C, Mei Z, Zhenping Z, Zhengjing H, Xiao Z (2019). Study of the prevalence and disease burden of chronic disease in the elderly in China. Chin J Epidemiol.

[13] Mcmullan M, Jones R, Lea S (2010). Patient safety: numerical skills and drug calculation abilities of nursing students and Registered Nurses. J Adv Nurs.

[14] Zurlo A, Zuliani G (2018). Management of care transition and hospital discharge. Aging Clin Exp Res.

[15] Wang SY, Zhao Y, Zang XY (2014). Continuing Care for Older Patients During the Transitional Period. Chin Nurs Res.

[16] Oikonomou E, Page B, Lawton R, Murray J, Vincent CJBHSR (2020). Validation of the Partners at Care Transitions Measure (PACT-M): assessing the quality and safety of care transitions for older people in the UK. BMC Health Serv Res.

[17] Coleman EA, Boult C (2010). Improving the Quality of Transitional Care for Persons with Complex Care Needs: Position Statement of The American Geriatrics Society Health Care Systems Committee. J Am Geriatr Soc.

[18] Li J, Brock J, Jack B, Mittman B, Naylor M, Sorra J (2016). Project ACHIEVE – using implementation research to guide the evaluation of transitional care effectiveness. BMC Health Serv Res.

[19] Burke RE, Hess E, Barón A, Levy C, Donzé J (2018). Predicting Potential Adverse Events During a Skilled Nursing Facility Stay: A Skilled Nursing Facility Prognosis Score. J Am Geriatr Soc.

[20] Francesca C, Susanna S, Silvia M, Elena L, Gabriele M, Luigi LP (2017). Unplanned readmissions within 30 days after discharge: improving quality through easy prediction. Int J Qual Health Care.

[21] Schectman JM (2013). ACP Journal Club. Review: Interventions for patient transition from hospital to primary care may improve outcomes. Ann Intern Med.

[22] Tully AP, Hammond DA, Li C, Jarrell AS, Kruer RM (2019). Evaluation of Medication Errors at the Transition of Care From an ICU to Non-ICU Location. Crit Care Med.

[23] Laugaland K, Aase K, Barach P (2012). Interventions to improve patient safety in transitional care-a review of the evidence. Work..

[24] Cao X, Lin C, Diao Y, Tian L, Liu W, Jiang X (2015). Validity and Reliability of the Chinese Version of the Care Transition Measure. PLoS One.

[25] Neale G, Woloshynowych M, Vincent C (2001). Exploring the causes of adverse events in NHS hospital practice. J R Soc Med.

[26] Io M (2006). Performance measurement: accelerating improvement.

[27] Organizations JCoAoH (2004). Shared Visions–New Pathways Q&A.

[28] Organizations JCoAoH (2006). National patient safety goals: 2006 critical access hospital and hospital national patient safety goals.

[29] Bakshi AB, Wee SL, Tay C, Wong LM, Leong YO, Merchant RA (2012). Validation of the care transition measure in multi-ethnic South-East Asia in Singapore. BMC Health Serv Res.

[30] Malley A, Kenner C (2016). Transitions in Care a Critical Review of Measurement. J Perioper Crit Intensive Care Nurs.

[31] Doyle C, Lennox L, Bell D (2013). A systematic review of evidence on the links between patient experience and clinical safety and effectiveness. BMJ Open.

[32] Howard-Anderson J, Busuttil A, Lonowski S, Vangala S, Afsar-Manesh N (2016). From discharge to readmission: Understanding the process from the patient perspective. J Hosp Med.

[33] Coleman EA, Mahoney E, Parry C (2005). Assessing the quality of preparation for posthospital care from the patient's perspective: the care transitions measure. Med Care.

[34] Masters S, Giles L, Halbert J, Crotty MJAJoA. (2010). Development and testing of a questionnaire to measure older people's experience of the Transition Care Program in Australia. Australas J Ageing.

[35] Boge MR, Haugen SA, Nilsen MR (2018). Elderly patients' (≥65 years) experiences associated with discharge; Development, validity and reliability of the Discharge Care Experiences Survey. PLoS One.

[36] Heather H, Henry B, Donald S, Michelle BF, Jennifer J (2008). Patient perceptions of hospital discharge: reliability and validity of a Patient Continuity of Care Questionnaire. Int J Qual Health Care.

[37] Uijen AA, Schellevis FG, Bosch WJHMvd, Mokkink H, Weel CV, Schers HJ. (2011). Nijmegen Continuity Questionnaire: Development and testing of a questionnaire that measures continuity of care. J Clin Epidemiol.

[38] Maria F, Mesfin T, Smstuen MC, Marléne L, Coleman EA, Mirjam E (2018). Measuring care transitions in Sweden: validation of the care transitions measure. Int J Qual Health Care.

[39] Efrat S, Anna Z, Coleman EA (2009). Translation and validation of the Care Transition Measure into Hebrew and Arabic. Int J Qual Health Care.

[40] Devellis RF (2016). Scale Development: Theory and Applications. Fourth ed. United Kingdom.

[41] Briggs SR, Cheek JM (1986). The Role of Factor-Analysis in the Development and Evaluation of Personality-Scales. J Pers.

[42] Brislin RW (1970). Back-Translation for Cross-Cultural Research. J Cross-Cult Psychol.

[43] Davis LL (1992). Instrument review: Getting the most from a panel of experts. Appl Nurs Res.

[44] Polit DF (2014). Getting serious about test–retest reliability: a critique of retest research and some recommendations. Qual Life Res.

[45] Roderick P, McDonald. (2011). The dimensionality of tests and items. British Journal of Mathematical Statistical. Psychology..

[46] Polit DF, Beck CT, Owen SV (2007). Is the CVI an acceptable indicator of content validity? Appraisal and recommendations. Res Nurs Health.

[47] Shrestha N (2021). Factor Analysis as a Tool for Survey Analysis. Am J Appl Math Stat.

[48] Polit DF, Beck CT (2010). The content validity index: are you sure you know what's being reported? Critique and recommendations. Res Nurs Health.

[49] Yingrui Y, Chengli W (2015). Reliability and validity of Chinese version of Heart Continuity of Care Questionnaire. Chin J Pract Nurs.

[50] Terwee C, Bot S, Boer M, DlAWMvd W, Knol DL, Dekker J (2007). Quality criteria were proposed for measurement properties of health status questionnaires. J Clin Epidemiol.

[51] Paiva CE, Barroso EM, Carneseca EC, Cristiano D, Santos F, López R (2014). A critical analysis of test-retest reliability in instrument validation studies of cancer patients under palliative care: a systematic review. BMC Med Res Methodol.

